# Medical Opinion and Movement

**Published:** 1907-11-02

**Authors:** 


### Medical Opinion and Movement.

Recent researches have indicated the possibility that the ordinary housefly may prove to be a formidable enemy as a carrier of the germs of disease. Dr. Alfred Kirchenberger and Herr Vala have recently been carrying out some observations upon a peculiar endemic gastric catarrh which occurs during the summer months in the southern provinces of Austria-Hungary. The disease attacks chiefly people in the district other than the natives, and falls especially heavily upon the younger soldiers in the barracks. The disease is characterised by fever, with severe headache and constipation. There is marked tenderness over the gastric region and a characteristic injection of the conjunctiva. Cramps in the muscles are common. An exanthem may occur resembling either urticaria or erythema multiforme. The condition lasts two to four days, and ends by crisis. By studying the distribution of the cases in the rooms of the barracks at Görz these observers have formed the opinion that the disease is communicated by the bite of the bug. It appears that these particular barracks are very old and are infested with bugs; whereas the other barracks, presumably cleaner, have remained free from the disease.

Although the beneficent effect of rest and fresh air in the treatment of the insane has been recognised by many authorities, and has, we believe, been practised to a greater or less extent in several asylums, it has not received any general adoption in the same manner as it is now employed for tuberculosis. Dr. C. C. Easterbrook's paper delivered before the International Congress of Psychiatry and Neurology at Amsterdam on the sanatorium treatment of active insanity by rest in bed and open air has aroused considerable interest. His experience of the treatment at the Ayr District Asylum, of which he is medical superintendent, has convinced him of its therapeutic value. All newly-admitted patients, unless too weak physically to be moved, are placed straightway in the verandahs attached to the reception wards of the hospital, where they remain throughout the day. The severity of the mental symptoms is no contra-indication to the treatment. According to this authority, there is a rapid subsidence of the active mental and nervous symptoms, and the improvement in the sleeping power and general physical condition of the patient is striking. The condition of the skin improves, the appetite recovers, and the increase in body-weight commonly amounts to from five to seven pounds during the first three weeks. No doubt Dr. Easterbrook's advocacy of the treatment will give rise to more extended trials in other quarters, and its value will then be more thoroughly tested and proved.

It may be said that in no branch of science has there been greater progress in recent years than in that of synthetic chemistry, and one may certainly add that no other advances in science have contributed more to the practical service of mankind or given greater assistance to other branches, to wit biology and physiology. Many industries owe their very existence to the synthetic chemist, and pharmacology has been much enriched by his products. This progress is the more remarkable to follow inasmuch as it has not been due to any sudden and dramatic revelation or discovery, but has been brought about by the steady, arduous work of many earnest labourers in the field. Among them Professor Emil Fischer occupies a prominent position, and his review of the subject in his Faraday lecture before the Chemical Society is consequently highly interesting and instructive. While describing the many successes which have recently been scored in building up complex organic compounds, he pointed out the inferiority of our laboratory methods in comparison with those of Nature. The chemist and the physiologist alike are met at every turn with the mystery of the ferment. Although the number of known ferments has increased to an extraordinary extent, yet we know hardly anything of their composition, and not a single one has as yet been definitely isolated. As Professor Emil Fischer remarked, the physiological chemist will concentrate his efforts more and more upon the study of fermentation changes. In our present vision a knowledge of the nature of these ferments would seem to offer a master-key that would unlock many doors and yield us a power to imitate the chemical processes of Nature such as we cannot possibly hope otherwise to attain.

By the kindness of the editor of *The Temperance Record*, which is the organ of the National Temperance League, we have received an account of a new and, in our opinion, a most praiseworthy departure of the executive of the League. Put quite shortly, the League has made up its mind that the scientific data at present available for accurate deduction with regard to the action of alcoholic beverages upon the human organism require considerable supplementing. Some four months ago we made up our mind in an exactly similar manner, and in a leading article in The Hospital of about that date we drew attention to the paucity of fact and to the plethora of theory upon this subject. We are quite sure that what the National Temperance League does, it will do well, and we hope that its research scholars will fully appreciate what an important issue hangs upon their researches, and will allow no bias, however philanthropical and well meant, to influence the deductions they draw from the scientific facts they ascertain. We do not wish for one moment to impute any anti-alcoholic preconceptions to any of those workers which we understand are to collaborate in this research, but at the same time apparently, to judge at least from recent literary productions, the most distinguished scientific workers, imbued doubtless with the best of motives, have allowed anti-alcoholic prejudice, not to say fanaticism, to colour the manner in which they have presented the literature of the alcoholic question to the public. We shall await with great interest the result of these investigations. The programme of subjects to be worked upon is most thorough and exhaustive, and the result when complete will certainly be a most important addition to pharmacological literature.

